# Development of eConsult reflective learning tools for healthcare providers: a pragmatic mixed methods approach

**DOI:** 10.1186/s12875-022-01948-9

**Published:** 2023-01-16

**Authors:** Douglas Archibald, Rachel Grant, Delphine S. Tuot, Clare Liddy, Justin L. Sewell, David W. Price, Roland Grad, Scott A. Shipman, Craig Campbell, Sheena Guglani, Timothy J. Wood, Erin Keely

**Affiliations:** 1 Department of Family Medicine, C.T. Lamont Primary Health Care Research Centre, University of Ottawa, Ottawa, Canada; 2 Bruyère Research Institute, Ottawa, Canada; 3 Faculty of Education, University of Ottawa, Ottawa, Canada; 4 Department of Medicine, Division of Nephrology, Zuckerberg San Francisco General Hospital, University of California San Francisco, San Francisco, CA USA; 5 Ontario eConsult Centre of Excellence, The Ottawa Hospital, Ottawa, Canada; 6 Department of Medicine, Division of Gastroenterology, Zuckerberg San Francisco General Hospital, University of California San Francisco, San Francisco, CA USA; 7 Department of Family Medicine, University of Colorado Anschutz School of Medicine, Aurora, CO USA; 8 The American Board of Family Medicine, Lexington, KY USA; 9 Department of Family Medicine, McGill University, Montreal, Canada; 10 Association of American Medical Colleges, Washington, DC USA; 11 Creighton University, Omaha, NE USA; 12 Department of Medicine, University of Ottawa, Ottawa, Canada; 13 Department of Innovation in Medical Education, University of Ottawa, Ottawa, Canada

**Keywords:** Electronic consultations, Continuing professional development, Delphi method, Mixed methods

## Abstract

**Background:**

Electronic consultation (eConsult) programs are crucial components of modern healthcare that facilitate communication between primary care providers (PCPs) and specialists. eConsults between PCPs and specialists. They also provide a unique opportunity to use real-world patient scenarios for reflective learning as part of professional development. However, tools that guide and document learning from eConsults are limited. The purpose of this study was to develop and pilot two eConsult reflective learning tools (RLTs), one for PCPs and one for specialists, for those participating in eConsults.

**Methods:**

We performed a four-phase pragmatic mixed methods study recruiting PCPs and specialists from two public health systems located in two countries: eConsult BASE in Canada and San Francisco Health Network eConsult in the United States. In phase 1, subject matter experts developed preliminary RLTs for PCPs and specialists. During phase 2, a Delphi survey among 20 PCPs and 16 specialists led to consensus on items for each RLT. In phase 3, we conducted cognitive interviews with three PCPs and five specialists as they applied the RLTs on previously completed consults. In phase 4, we piloted the RLTs with eConsult users.

**Results:**

The RLTs were perceived to elicit *critical reflection* among participants regarding their knowledge and practice habits and could be used for quality improvement and continuing professional development.

**Conclusion:**

PCPs and specialists alike perceived that eConsult systems provided opportunities for *self-directed learning* wherein they were motivated to investigate topics further through the course of eConsult exchanges. We recommend the RLTs be subject to further evaluation through implementation studies at other sites.

**Supplementary Information:**

The online version contains supplementary material available at 10.1186/s12875-022-01948-9.

## Background

Relevant, active, reflective learning in workplaces is challenging for health professionals, requiring clinicians to have metacognitive skills and the ability to learn opportunistically [1]. One example of a workplace learning activity is provider to provider electronic consultations (eConsults), a type of asynchronous communication whereby primary care providers (PCPs) and consultant specialists communicate using a secure electronic platform, enabling transfer of information from one healthcare provider to another. eConsults facilitate iterative communication until the PCP’s question has been answered or the patient’s issue has been addressed. The primary intent is to provide efficient, timely access to specialty expertise, reduce the need for unnecessary face-to-face or virtual specialty consultations, and improve the quality and efficiency of the initial face-to-face consultation through pre-visit communication involving diagnostic or treatment advice [2, 3]. While eConsults can potentially foster reflection [4] little is known about their actual impact on learning. eConsults provide an opportunity to link learning to practice, promote greater reflection on practice, and document learning among both PCPs and specialists.

Continuing professional development needs to align with continuous quality improvement initiatives in the workplace in order to enhance the quality and safety of health care delivery [5]. Greater emphasis on learning “from practice” and “in practice” requires assessment processes that provide data on competence or performance with feedback. Clinical questions captured by eConsult systems are generated from practice. Thus eConsult interactions can provide evidence of self-monitoring by PCPs as they self-identify when they need to slow down and learn before acting [6]. They can also identify when Specialists engage in self-monitoring, when crafting a clinical response to the PCP, perhaps requiring consultation of recent medical literature or an expert colleague. eConsults can serve as self-monitoring tools for both PCPs and specialists, with output potentially incorporated into longitudinal portfolios of individual learning activities to document lifelong learning and self-assessment for maintenance of certification or continuing medical education programs [7]. However, current tools that guide and document learning from eConsults are limited. In this study, we developed, tested, and piloted two eConsult reflective learning tools (RLTs), and identified elements and processes that facilitate the use of eConsults as a means of reflective CPD.

## Methods

### Pragmatism

The underlying philosophy of this study is pragmatism, which is often recommended in mixed methods research [8–11]. Pragmatism has been described as a “question-driven philosophy” [12] where the research question is central to the research process, impacting subsequent decisions about methodology and methods [13]. It considers how different approaches will impact the results, as well as what it means to produce one kind of knowledge over others [9]. It also gives researchers the freedom to select the most appropriate methodology and methods to answer their research question, liberating researchers from false senses of loyalty to particular epistemological or research traditions [10, 14]. Pragmatism does not restrict itself to constructive or realist understandings of reality that may be garnered from qualitative or quantitative research respectively [9] Pragmatism does not focus on ontological arguments about the nature of reality, and accepts there are multiple realities rooted in individuals’ own interpretations of the world [9, 13]. It addresses any ontological and epistemological incommensurability by introducing a paradigm that allows for flexibility, and focuses on the utility of outcomes when addressing the research question [10, 15, 16].

### Theoretical foundation

**Fig. 1:**
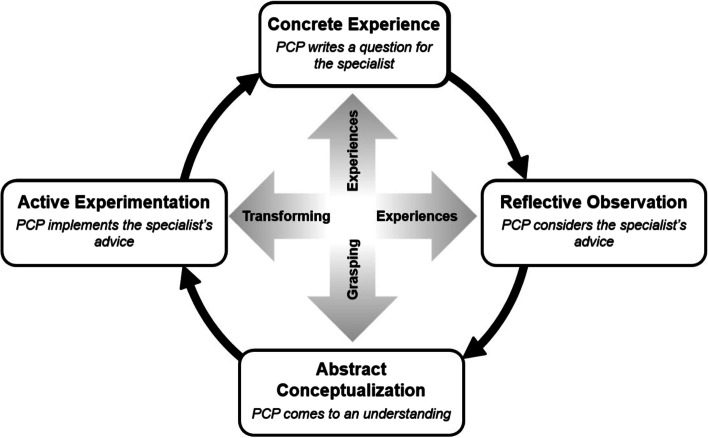
Application of the Kolb learning cycle to eConsults in the context of a specific patient’s care [4]

The theoretical foundation of this study is driven by reflective learning and the application of the Kolb Learning Model. *Reflective practice* was coined by Schön in the 1980s, defined by contemporary scholars as “a way of practicing, emphasizing processes of professional consideration – based on multiple sources and concepts of knowledge – before, after and in the midst of professional actions.” [17]. Kolb’s experiential learning model juxtaposes reflective practice (a way of practicing, emphasizing processes of professional consideration) with active experimentation (testing implications of the reflectively and abstractly acquired understandings of concrete experience to make decisions and solve problems [18]. This theory frames PCPs’ reflective learning from eConsults (Fig. 1) [4, 19]. The cycle is recursive, beginning anew with each eConsult.

### Study design

**Fig. 2:**
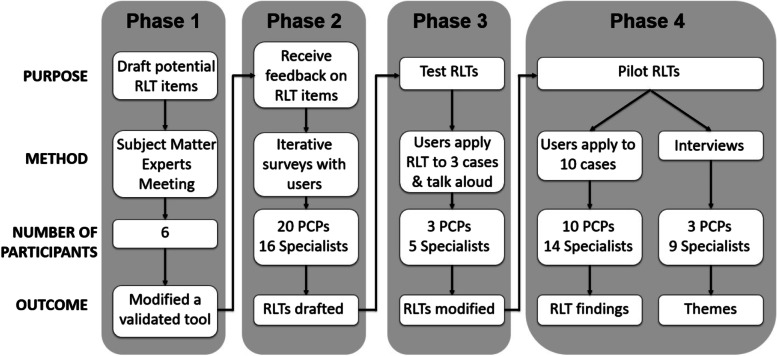
Overall research design

A mixed methods approach allows researchers to construct knowledge that cannot be produced solely from qualitative or quantitative research, and can lead to more complex, in-depth understandings of the phenomena from multiple perspectives [8, 10, 20, 21]. We used a pragmatic multiphase mixed methods design with four sequential phases to garner an in-depth, comprehensive understanding of learning that occurs within eConsult exchanges (Fig. 2).

In Phase 1, subject matter experts (henceforth referred to as experts) met to draft potential RLT items, which were then further refined by the research team. Subsequent drafts were reviewed and refined by 36 eConsult users in Phase 2 using the Delphi method, which utilized Likert scales and free-text comments. The research team considered and incorporated both the quantitative and qualitative data from Phase 2 into the RLTs prior to testing them. In Phase 3, eight eConsult users tested the draft RLTs on three of their own eConsult cases and participated in a 30-min cognitive interview about their experience. The RLTs were further refined before being piloted in Phase 4 wherein ten eConsult users piloted the two RLTs for a nine-month period, and then participated in a 30–60 min semi-structured interview. Results from Phase 4 – including themes from the interviews and experience using the RLTs in real time– were linked back to findings in Phase 2 and 3.

The Bruyère Continuing Care Research Ethics Board (REB) (protocol #M16-17–021) and the Ottawa Health Science Network REB (protocol #20,170,057-01H) reviewed and approved this project.

### Setting

We chose two well-developed multi-specialty asynchronous eConsult programs for this study: eConsult BASE (Building Access to Specialists through eConsultations) from Ontario, Canada, and San Francisco Health Network (SFHN) eConsult from California, USA. eConsult BASE is a Canadian eConsult service that uses a secure web-based platform with a standardized electronic form through which—primary care physicians and nurse practitioners (in this paper collectively referred to as PCPs) can submit non-urgent patient-specific clinical queries to a wide range of specialists [22]. It was developed in 2010 in the Champlain Local Health Integrated Network (LHIN) in Ottawa, Ontario to reduce wait times and improve access to specialist care [23]. The Champlain LHIN serves approximately 1.3 million people who live in Ottawa and its surrounding communities [24], and in 2019, at the time of data collection, encompassed 3,860 physicians (1,777 family physicians and 2,083 specialists) and 399 nurse practitioners (NPs) [25]. Since its inception, the eConsult BASE service has grown to include 153 specialty groups in 105 communities and the 100 000^th^ eConsult was completed in December 2021 [26, 27].

The SFHN eConsult is an integrated eConsult and referral system that is used across its provider network, which serves approximately 123,000 uninsured and underinsured residents in San Francisco [28, 29]. Unlike eConsult BASE – which is centered around a clinical question posed by the PCP in the absence of an expected face-to-face visit – SFHN eConsult is a mandatory pathway for referring providers to request non-emergent specialist expertise [30]. A PCP submits an eConsult through the platform, which is then sent to a reviewer for that specialty [28]. The reviewer can be a physician or an advanced care provider with physician oversight (e.g., NP or physician assistant). After careful review, the specialist notes whether or not the patient would benefit from a face-to-face appointment, and responds to the referring provider with consultative diagnostic or management advice including in the timeframe in which they are requesting to schedule face-to-face specialty visit, if appropriate [30]. Nearly 600,000 eConsults have been completed since 2005 submitted by 6,935 providers. In both systems, all referral questions and specialist replies are accessible for analysis.

### Phase 1: drafting the RLTs

#### Identifying existing reflective learning instruments

We identified two existing instruments for reflective learning within health professions education. The IAM Clinician tool has been used since 2006 to stimulate and guide reflective learning for documentation of CPD [2, 31]. It is comprised of the IAM Clinician Search, a questionnaire that guides users to evaluate information retrieved from a knowledge resource (also known as the *pull* context); and the IAM Clinician Push, a similar questionnaire delivered to the user via email [2, 3, 31]. The second instrument we identified was developed by author DP. This 13-question reflection tool asks participants to reflect on the impact of a quality improvement activity on their clinical and operational work, and whether changes were made as a result within their clinical team. If changes were made, the participant is asked what changes were made and tips for success for other teams. If not, they are asked to reflect on the barriers to making change (e.g., cost, organization priorities, or the skill, training and time of staff). Narrative questions prompt the individual to reflect on their participation, such as what went well, areas for improvement, next steps, and potential barriers to ongoing and sustained improvement [32]. Versions of the form have been used at CPD activities in large health systems and for quality improvement activities submitted for maintenance of certification credit through the ABMS.

#### Expert meeting

We held the meeting in October 2017 in Ottawa, Ontario, which included clinical and methodological experts [33]. Participants included members of the project team (American and Canadian researchers with backgrounds in medical education, epidemiology, medicine, nursing, and psychometrics) and six users spanning both eConsult systems. Following an overview of the study methods, the team reviewed existing RLTs and discussed their applicability to eConsults. The experts selected IAM Search and IAM Push as the starting point for developing the RLTs, with the understanding that modifications would be made to tailor it to the eConsult context.

### Phase 2: refining the RLTs

Based on Phase 1, we developed two RLTs; a PCP RLT and a specialist RLT. In Phase 2, we sought feedback from active eConsult users and further refined the drafted RLTs into prototypes that could be tested in Phase 3 using the Delphi method.

### Data collection

The Delphi method is a consensus group method that uses iterative anonymous surveys whereby experts rate or rank items until consensus is reached [34, 35], or after a predetermined number of survey rounds [35]. In this study, we used iterative electronic surveys to work towards consensus on which proposed items/questions should be included in the RLTs.

The Delphi portion of this study consisted of input from 36 participating eConsult users on whether to include proposed RLT items. The surveys were drafted by the Project Manager and Principal Investigator, and programmed into Survey Monkey™. For each RLT item, participants were asked to use a five-point Likert scale to rate: *How important is it to include or exclude this question in the reflection tool?* Response options were *definitely include*, *possibly include*, *neutral*, *possibly exclude*, and *definitely exclude*. Participants could leave optional free-text comments after each question and at the end of the survey. Participants had one week to complete each survey. Those who did not respond to a survey were unable to progress to the next survey round.

In follow-up surveys, we sent participants an individual report which contained the aggregate results with their responses highlighted [36] and the matrix tables summarizing the free-text comments. We asked participants to review their previous responses and those of their colleagues before responding to the survey. This allowed participants to reflect on their initial judgements prior to responding to the subsequent survey [37]. We anticipated two or three Delphi rounds, as commonly reported in the literature [34, 35]. Our team decided that the surveys would continue beyond two rounds if there remained substantial movement amongst response options, but would be stopped once consensus was reached on all of the items or stability in the response options was reached regardless of consensus [34]. Consensus was defined as 70% on a single point, or 80% or greater on two adjacent points (for example, *definitely include* or *possibly include)* at either end of the 5-point Likert scale [36, 38].

#### Analysis

Analysis included descriptive statistics reporting on the responses for each potential RLT item. Post-hoc analysis compared the responses between countries and professional groups for each RLT. We analyzed free-text comments using a conventional content analysis approach to identify themes [39]. To preserve anonymity, only the Project Manager knew the identities of those who wrote the comments. The Principal Investigator was able to view anonymized versions of the comments. The frequency at which each theme occurred was determined and these themes along with their frequency were displayed in matrix tables ranked from most to least common [40, 41].

### Phase 3: Testing the RLTs

In Phase 3, we tested the feasibility of using the RLTs to document learning. This testing allowed the research team to refine the number of items on the RLTs and modify the wording of items.

#### Data collection

eConsult users registered as PCPs or specialists in the eConsult BASE service or SFHN eConsult were invited to participate. We aimed to recruit six PCPs and six specialists from each service. We programmed the RLTs into Survey Gizmo and tested with the research team prior to testing by eConsult users. We asked participants to apply the RLTs to three recent eConsult cases of their own – preferably one that was easy, one that was more complex, and one in between – and talk aloud as they answered the RLT questions. We encouraged participants to express their thoughts – such as questions being unclear – as they answered it.

During these think-aloud sessions, a method used in social science and educational research to generate verbal response reports for analysis [42], the observing researcher took notes on what the participants said, as well as including their own observations. The findings were shared with the research team to decide if further modifications to the RLT were needed.

#### Analysis

The analysis consisted of reviewing the observing researcher’s notes on what the participants said and their own observations during the think-aloud sessions. To preserve anonymity, only the observing researcher knew the identities of those who wrote the comments.

### Phase 4: piloting the RLTs

We piloted the RLTs from January—September 2019 to gather data on the effectiveness of the RLTs when completed at the end of an eConsult exchange. All PCP and specialist users of eConsult BASE and SFHN eConsult were invited to participate in the pilot of the RLTs. Invitations were sent out via email by members of the research team. We asked participants to complete the RLT at the end of each eConsult case during the pilot phase, and then participate in an interview. The RLTs were programmed into Survey Gizmo. For PCPs, the PCP RLT survey link was sent when they closed an eConsult case. Specialists were sent the specialist RLT survey link when they submitted their response to the PCP. At the end of the pilot, we analyzed RLT submissions which informed the interview questions. The survey links were sent to consenting participants by a member of the research team.

#### Interviews

We invited pilot participants to participate in an interview after they completed ten RLTs as an estimate of a provider’s usual practice. The research team developed a semi-structured interview guide to solicit their perceptions of their experience using the tools, including potential improvements and their thoughts on using the RLT as a means to give feedback to PCPs or responding specialists. We also asked participants about their perceptions of learning from eConsults and the building of trust or rapport through eConsult dialogues. Questions were open-ended to avoid inadvertently imposing a particular response and to elicit rich descriptions [43]. We anticipated interviews to last about 60 min. All interviews except one were conducted by telephone. Three members of the research team were responsible for conducting interviews and transcribing their own interviews.

#### Analysis of RLT responses and transcripts

We downloaded reports on the RLT submissions from both eConsult BASE and SFHN eConsult from Survey Monkey. We analyzed the submissions using descriptive statistics.

Transcripts were imported into NVivo for analysis. The research team performed an inductive thematic analysis of the transcripts [39]. Multiple researchers were involved in this process to increase interpretive rigour [44]. The three interviewers developed an initial coding framework by individually coding two transcripts and then meeting to discuss their findings. As a group, they went through both transcripts and their proposed coding until consensus on codes was reached, which comprised the initial coding framework. The coding framework along with the two transcripts were sent to a fourth member of the research team to code and suggest changes. The coding framework was then refined and shared with the rest of the research team for final modifications. The research team was then able to apply this coding framework to the remainder of the transcripts.

## Results

Since this mixed methods study used sequential phases, the findings of each phase provided the foundation for the subsequent phase. As such, the study’s findings are organized by phase, as described below.

### Phase 1: drafting potential RLT items

IAM Push was selected as the foundation for the PCP RLT because PCPs receive information from the specialist. IAM Search was selected as the foundation for the specialist RLT as we had anecdotal evidence that specialists sometimes consult other resources and colleagues when presented with more difficult eConsult cases. Both questionnaires were modified to be more amenable to reflective learning through eConsults. Modifications to IAM Push included changes in phrasing and additional questions, such as the type of clinical question and the main learning point(s). IAM Search was modified to include feedback to the PCP, information on the resources consulted, and main learning point(s). In both questionnaires, the question on ‘Application to patient care’ was retained but shortened. Discussion regarding these modifications began during the expert meeting and continued online amongst members of the project team until the first iteration of the RLTs was finalized.

### Phase 2: refining the RLT items

A total of 36 participants (18 American users of SFHN eConsult and 18 Canadian eConsult BASE users), participated in the Delphi surveys. Response rates at each stage of data collection for each survey are shown in Table 1. Over the course of the Delphi surveys, we lost 7 PCPs (3 NPs and 4 MDs) and 3 specialists (all MDs) due to non-response.

**Table 1:** Response rates for each survey round

**Delphi Round**	**Groups** **Primary Care Providers** ***n* = 20; Specialists *n* = 16**	**After Initial Mail Out, %**	**After First Reminder, %**	**After Second Reminder, %**
1	Primary Care Providers	40	60	90
	Specialists	63	81	100
	Overall response rates	50	69	94
2	Primary Care Providers	50	72	89
	Specialists	56	69	94
	Overall response rates	53	71	91
3	Primary Care Providers	50	69	81
	Specialists	53	67	87
	Overall response rates	52	68	84

We completed three rounds of the Delphi survey over a four-month period. The research team received a summary of the quantitative (percentage of consensus for each question) and qualitative (comments from participants) results after each Delphi iteration to discuss and modify as necessary before sending out the survey to the participants. By the third round, either consensus or stability had been reached for most items. All unresolved items were leaning towards consensus, and concerns raised in the comments largely revolved around feasibility in practice. The team decided to include these items in the RLTs and seek feedback from participants during Phase 3.

20 PCPs participated in the Delphi process. The overall response rates for the surveys were 90% (round 1), 88.9% (round 2), and 81.3% (round 3). After three rounds of surveys, PCPs reached consensus to include 3 out of 7 items. 16 specialists participated in the Delphi process. The overall response rates for the surveys were 100% (round 1), 93.8% (round 2), and 86.7% (round 3). After three rounds of surveys, specialists reached consensus to include 3 out of 6 items. Additional Files 1 and 2 depict the Delphi results for each RLT. Table 2 shows the final items and questions after completion of the Delphi.

**Table 2:** Final PCP and Specialist RLT items and questions

**Primary Care Provider RLT**		
**No**	**Item**	**Question**
1	Clinical Question	What was your clinical question?
2	Learning	How did the special’s response to this eConsult impact your knowledge or understanding?
3	Improvement to the Specialist’s Response	How could this response have been improved?
4	Application to Patient Care	Will you use this eConsult information for your patient?
5	Anticipated Benefits	Do you expect any benefit(s) to the patient as a result of applying this eConsult information?
6	Sharing Patient Outcomes	If you and this patient are willing to share the patient outcomes with the specialist, please click here
7	Send Feedback to the Specialist	Are you willing to share a copy of this survey with the specialist?
**Specialist RLT**		
**No**	**Item**	**Question**
1	Clinical Question	Was the clinical question clear?
2	Information to Facilitate Consultation	Did the PCP include sufficient and appropriate information to facilitate your consultation?
3	Resources Consulted	What resources did you use to answer the PCP’s question other than personal knowledge/experience?
4	Additional Information from PCP	Did you seek additional information from the referrer?
5	Learning & Application of Learning	Did you learn anything from this eConsult request? How are you planning to use this information in your practice?
6	Sharing Patient Outcomes	Would you like the PCP to share the patient outcomes with you?

### Phase 3: testing the RLTs

#### Results for the PCP RLT

Three primary care physicians (two from SFHN and one from eConsult BASE) participated in testing the PCP version of the RLT. Each RLT took providers up to a maximum of five minutes to describe.

Two PCP participants noted a specialist will sometimes give guidance but not explain the underlying rationale. They commented that while the advice given by the specialist may result in a change in practice, in such instances they did not feel they were learning. One of the SFHN PCPs commented that some specialists are more likely to explain their reasoning than others who just give instructions. While reviewing one case, this provider commented, “[They] mainly gave me additional labs. More so told me what to do without a lot of information about why… Maybe it’s not much of a learning experience”.

This participant also stated that she could have managed more if she had been given more information by the specialist. This led our team to posit that eConsults may be more of a learning experience for the PCPs when the specialist is acting as a knowledge interpreter. The research team decided this was something that warranted further exploration in Phase 4.

#### Results for the specialist RLT

Five specialists (one NP and four physicians) participated in testing the specialist version of the RLT, including two SFHN eConsult users and three eConsult BASE users.

One theme that emerged from the testing was that *specialist learning from eConsults is not confined to matters of medical expertise*. Many of the specialists seemed to struggle with what it meant to learn from an eConsult. One participant stated that “Maybe learning isn’t quite the right word”. Another commented that “Maybe there needs to be something about reflecting on themselves [the specialist’s role] as an educator in the eConsult interaction”. The project team decided this was something that needed to be explored in more depth in Phase 4.

A second theme was reassurance: two specialists indicated that many of their eConsult cases involve simply reassuring the PCP. A specialist with eConsult BASE said that for these cases he often does not have to look anything up. They’re mostly about reassurance and education so they [the PCP] can manage it locally,” he explained. The research team noted that this overlapped with *the specialists as knowledge interpreters* theme from the PCP RLT testing, and warranted further exploration in Phase 4.

### Phase 4: piloting the RLTs

#### PCP RLT findings

Participants included one NP and five physicians with the eConsult BASE service and one NP and three physicians with the SFHN eConsult service. Sixty RLTs were completed, 29 submitted by eConsult BASE users and 31 by SFHN eConsult users. Additional File 3 presents an algorithm of the PCP RLT items and response options and represents the final version of the tool.

##### PCP RLT item 1: Clinical question

We asked participants to identify the type of clinical question they submitted. Participants could select more than one response option. The most common clinical question types were *clinical management* (51.6%, 31/60) and *diagnostic* (51.6%, 31/60). Questions pertaining to *treatment* were less commonly reported (33.3%, 20/60).

We asked participants to identify the specialty they consulted. Overall, the most common were internal medicine specialties (45.0%, 27/60), surgical specialties (26.7%, 16/60), and dermatology (11.6%, 7/60). The list of services consulted in this study can be found in Additional File 4.

##### PCP RLT item 2: learning

We asked participants how the specialist’s response to this eConsult impacted their knowledge or understanding. Participants could select more than one response option. PCPs more commonly reported they *learned something new* (51.7%, 31/60). When a participant indicated they learned something new, they were asked to list at least one learning point. For example, “Gastric resection can have an impact on iron stores that may contribute to anemia” (SFHN eConsult physician). 23.3% (14/60) responded they were *motivated to learn more.* 28.3% (17/60) PCPs reported *feeling reassured;* (23.3%, 14/60) indicated the response *not impacting their knowledge and understanding.*

##### PCP RLT item 3: improvements to the specialist’s response

In 20% or 12/60 of RLT submissions, PCPs indicated the specialist’s response *could have been improved*. For example, clearer communication surrounding whether a referral was still needed.

##### PCP RLT item 4: application to patient care

We asked participants if they would use the eConsult information for their patient (*yes*, *possibly*, or *no)*. In 86.6% (52/60) of RLTs submitted, participants indicated they would use this information for their patient. The most commonly reported applications to patient care were that as a result of the eConsult they would *manage this patient differently* (41.7%, 25/60) and *use this information to justify a choice* (41.7%, 25/60).

##### PCP RLT item 5: anticipated benefits

We asked participants if they expected any benefit(s) to the patient as a result of applying this eConsult information. Participants were able to select all responses that applied. The most common anticipated benefit was *helping avoid an unnecessary or inappropriate treatment*, *diagnostic procedure*, *preventative intervention* or a *referral for this patient* (60%, 36/60).

##### RLT item 6: sharing patient outcomes

We asked participants if they and the patient were willing to share the patient outcomes with the specialist. In 30% (18/60) of RLTs, PCPs were willing to share patient outcomes.

##### PCP RLT item 7: sharing RLT copies with the specialist

68.3% (41/60) participants were willing to share a copy of their RLT with the specialist.

#### Specialist RLT findings

Participants included seven physicians with eConsult BASE and three advanced clinical professionals (two NPs and one physician assistant) and four physicians with SFHN eConsult. A total of 120 RLTs were completed: 58 submitted by eConsult BASE users and 62 by SFHN eConsult users. Additional File 5 displays an algorithm of the specialist RLT items and response options, and represents the final version of the tool.

##### Specialist RLT item 1: clinical question

A total of 120 RLTs were submitted. All RLTs submitted by eConsult BASE specialists (100%, n = 58/58) indicated the clinical question was *clear*. Of those who indicated the clinical question was *unclear*, two-thirds indicated they were *still able to provide an answer to the question*. Those who were unable to answer the clinical question did not progress to subsequent questions.

Of those that indicated the clinical question was *clear* (*N* = 105), the most commonly reported types of clinical questions were *clinical management* (65.7%, 69/105), *diagnostics* (52.4%, 55/105), and *treatment* (41.9%, 44/105).

##### Specialist RLT item 2: information to facilitate consultation

Overall, 80.2% (93/116) of RLTs that progressed to the second item indicated the PCP *included sufficient and appropriate information*.

Of those that indicated *insufficient or inadequate information* was included in the eConsult request (N = 23), nearly three-quarters (73.9%, 17/23) suggested this was because the *relevant clinical history was inadequate*.

##### Specialist RLT item 3: resources consulted

The majority of participating specialists (88.7%, 102/115) indicated they *did not consult an additional resource* to answer the PCP’s question. Specialists who consulted other resources often reported referring to a *clinical practice guideline* (38.5%, 5/13).

##### Specialist RLT item 4: additional information from the PCP

In 40% of RLTs, the specialist indicated that they *sought additional information from the referrer* (46/115) Participants most commonly *sought additional information* through their *eConsult platform* (91.3%, 42/46) or *additional review of the patient’s record* (19.6%, 9/46).

Clinicians most commonly indicated they sought out additional information to *clarify information about the patient* (65.2%, 30/46) or to *request additional diagnostic test results* (45.6%, 21/46).

##### Specialist RLT item 5: learning and application of learning

Overall, 26.1% (30/115) of RLTs submitted indicated the participating specialist had *learned something from the eConsult exchange*. Overall clinicians most commonly indicated they would use this information in their practice by *sharing it with other healthcare professionals* (63.3%, 19/30) or using the information in the *teaching of their trainees* (36.7%, 11/30).

##### Specialist RLT item 6: Sharing patient outcomes

Overall, clinicians in 67.5% of RLT submissions that progressed to RLT item six wanted the PCP to share the patient outcomes with them (27/40).

#### Interview findings

We interviewed a total of 12 clinicians, including three PCPs and nine specialists. While our initial plan was to interview them after they completed 10 RLTs, some were unable to reach the target number. For example, one PCP submitted three eConsults during the pilot phase, so only had three opportunities to complete the RLT. Nevertheless, these participants were contacted for an interview at the conclusion of the pilot. Analysis of the interview transcripts revealed the following themes: feedback, teaching and learning; and trust.

##### Feedback

Pertains to capturing providers’ perceptions of the giving and receiving of feedback with regards to eConsults between PCPs and specialists. Within this theme we have identified 6 subthemes: adequate information, anonymity, format, patient outcomes, improving the RLTs, and timing. Adequate information refers to specialists’ perceptions of the information they need from PCPs to facilitate an eConsult. Anonymity of giving feedback to other providers via the RLTs was an important consideration. Providers indicated preferred formats such as receiving a report or creating a dashboard as a potential source for quality improvement. Specialists made it clear that following-up on patient outcomes should be optional, understanding that PCPs are busy and will contact them if they are concerned. Providers thought a great improvement to the RLTs would be creating a mobile app which would make completing the RLT convenient and quick. In terms of timing, providers thought that receiving quarterly feedback summaries would be helpful.

##### Teaching and learning

This category pertains to participants’ perceptions of teaching and/or learning occurring through or because of eConsults. Within this theme we have identified 6 subthemes: communities of practice, self-regulated learning, critical reflection, practice patterns, scaffolding, and specialist as a knowledge translator. The notion of communities of practice surfaced during the interviews, in that providers learn from sharing information/experience and develop a shared repertoire of knowledge. Self–regulated learning was also a common subtheme, as interviewees cited examples of eConsults motivating them to seek out continuing professional development activities. There were numerous examples of critical reflection given by providers, which challenged them to change assumptions they had about practice. The following example is representative of many:

I think it was one or two questions that asked really specifically about Did you learn anything from this or Did you share – or really it was about Did you share any information back... And at the beginning I found I kept saying no, which got me thinking about well, why am I not doing that? Cause it’s not that the questions I get are very easy or just simple like scheduling a patient for a procedure. Many times it was more diagnostic or treatment questions. So really I had to pause and think about it in terms of, you know, how do I give information to individuals when they may be having questions? What framework can I do it in? I talked to one of my colleagues about it and we came up with this framework for how we wanted to move forward with it” (SFHN eConsult specialist)

PCPs commented that completing RLTs over time could allow one to see how practice patterns develop. Specialists providing support to a PCP via eConsult enabled the PCP to safely seek guidance until they are able to perform unaided, a term known as scaffolding [45]. The idea that the specialist was a knowledge interpreter was noticed as they share knowledge and evidence with PCPs via eConsult, so RLTs can be used in clinical practice.

##### Trust

This category pertains to participants’ perceptions of trust between PCP and specialist eConsult users. Within this theme we have identified 2 subthemes: in-person relationships, contributing to the establishment of trust between PCPs and specialists, the PCPs’ perceptions of specialist engagement in eConsults creating trust and rapport.

## Discussion

In this study, we developed and tested two eConsult RLTs for PCPs and specialists. We identified elements and processes that facilitate the use of eConsults as a means of reflective practice. Our findings reinforce the Kolb Learning Model as an effective model of experiential learning for eConsults, positing four learning abilities. First, PCPs are very willing to engage in eConsult programs as evidenced by the exponential increase in use in recent years, especially given rapid growth in virtual care [46]. Second, many participating providers in our study engaged in reflective observation by documenting their learning. Third, providers undertake abstract conceptualization, by coming to a mutual understanding for the best care management plans for the patients in question. Growing relationships between providers as clinical advice is being implemented by PCPs and specialists’ desires for feedback and sharing of patient outcomes aligns with the active experimentation phase of the Kolb model.

Feedback provided by participants through testing and piloting of the two RLTs, indicates promise for future use. The RLTs could be used to construct a learning database for clinicians to structure and document their self-learning through reflection both in-practice and on-practice. By completing eConsults using the RLTs, providers have a compendium of the consults and questions they have requested or addressed. This could help providers further reflect on questions they are asking or addressing, patient care and outcomes, and opportunities for prompting for learning. Similar findings were found in previous work on the educational value of eConsults [4, 47–49].

Although there was variation in the amount of learning demonstrated through eConsult exchanges by providers, the RLTs allow specialists to act as educators/knowledge interpreters by providing aids and support for providers as they navigate clinical questions and provide clinical advice to patients. Completing eConsults using the RLTs provides instructional scaffolding for those requesting consults, enhancing their learning until they can perform unaided [47]. It is paramount that clinicians think critically about the clinical advice they give or the implementation of the advice they receive for patient care. Educational strategies, such as the development of RLTs in this study, and rigorous designs to evaluate their effectiveness are needed to promote reflective practice and continuing professional development in the health professions. We will develop a mobile application, or modify an existing one, to facilitate the completion of RLTs and evaluate its effectiveness and usability as a mobile learning tool.

We found the feedback, and teaching and learning outcomes in this study are similar to other eConsult studies [19, 50]. eConsults support experiential learning [4], self-regulated learning, and critical reflection. This study contributes to work on communities of practice, as participating in eConsults and completing RLTs help to develop trust between PCPs and specialists. Through discourse and reflection, relationships are formed between providers and can lead to implications for educational practice support, the call for learning driven by individual clinical practice needs [4, 5, 47, 51].

A strength of the study is the use of pragmatism in the methodology to inform the development and testing of the RLTs across two eConsult systems. This epistemological approach allowed for flexibility in the development of the RLTs, focusing on the utility of the outcomes [10, 15, 16]. It facilitated ongoing discussions of how different approaches would impact the development of the RLTs, and allowed us to address concerns (such as feasibility in practice) as they arose [37].

Mixed methods research can be time consuming, and challenging as it may yield large amounts of data from different sources [51, 52]. To mitigate this, we assembled a large, active, interdisciplinary, methodologically diverse team, allowing for more in-depth and comprehensive discussion about the methodological approaches and data interpretations. Phase 2 (the Delphi Survey) became complicated. Providing participants with a flowchart or algorithm to view the flow of responses may have made the process less overwhelming. Several items in both RLTs did not achieve consensus, based on participant comments about the feasibility of completing the RLTs in practice, the research team kept many of these items in the RLTs and sought further feedback from participants during the testing stage.

One limitation of our study is low numbers of providers involved in all phases of the study. This could be perceived as a threat to the validity of the findings given the large number of clinicians using both eConsult platforms. However, this was chiefly a proof of concept study so were less concerned about generalizability at this stage. This said, we were purposeful in the invitations sent to providers to ensure a wide variety of PCPs and specialists across both platforms including nurse practitioners, physician assistants, and urban and rural representation. Further research is needed to assess the effect of these RLTs in other eConsult systems around the world and their learning implications for continuing professional development, helping clinicians to reflect and think critically about their practice, learning needs, and patient care. Further investigation is also needed to explore the effect of the RLTs for NPs and other advanced care providers.

## Conclusion

We have defined core elements and processes that facilitate the development of RLTs using eConsults as a means of promoting reflective learning in the clinical setting. The study findings support the potential use of RLTs as a continuing professional development activity for clinicians, promoting the self-reflection, instructional scaffolding, and critical reflection of clinical advice received and/or given to apply to patient care in a clinical setting.

## Supplementary Information

**Additional file 1. **Results of the three Delhi rounds for primary care providers’ reflective learning tool.

**Additional file 2. **Results of the three Delhi rounds for specialists’ reflective learning tool.

**Additional file 3. **PCP RLT Algorithm.

**Additional file 4.** Specialties or Services consulted by PCPs.

**Additional file 5. **Specialist RLT Algorithm.

## Data Availability

The data is stored on the Bruyère Continuing Care Shared Drive and is available upon request to the corresponding author Dr. Douglas Archibald, darchibald@bruyere.org.

## Abbreviations

eConsults: Electronic Consultations
RLT: Reflective Learning Tool
PCP: Primary Care Provider
NP: Nurse Practitioner
BASE: Building Access to Specialists through eConsultations
SFHN: San Francisco Health Network
LHIN: Local Health Integrated Network
IAM: Information Assessment Method
ABMS: American Board of Medical Specialties
