# Supplementary material for: Development of eConsult reflective learning tools for healthcare providers: a pragmatic mixed methods approach
Source: BMC Prim Care. 2023 Jan 16;24:15. doi: 10.1186/s12875-022-01948-9 (PMC9841624; doi:10.1186/s12875-022-01948-9)
Supplement: Supplementary file 1 — Additional file 1. Results of the three Delhi rounds for primary care providers’ reflective learning tool. [file 12875_2022_1948_MOESM1_ESM.pdf]

Additional File 1. Results of the three Delhi rounds for primary care providers' reflective learning tool.

Round 1

Seven items including open text boxes for comments

Tool Content

Item 1. What was your clinical question?
- Checkboxes and open text box to indicate specialty consulted

Item 2. How did the specialist's response to this eConsult impact your knowledge or understanding?
- Six response options (select all that apply)

Item 3. Could this response have been improved?
- Option yes or no; if yes then 6 response options (select all that apply)

Item 4. Will you use this eConsult information for your patient?
- Option yes, possibly or no
- If yes, five response options (select all that apply)
- If possibly, five response options (select all that apply)
- If no, five response options (select all that apply)

Item 5. Do you expect any benefit(s) to the patient as a result of applying this eConsult information?
- If yes, five response options (select all that apply)

Item 6. If you and this patient are willing to share the patient outcomes with the specialist, please click here.

Item 7. Are you willing to share a copy of this survey with the specialist?
- Option yes or no

→

Outcomes

Item 1. Consensus reached (76.5%)

Items 2-7. Consensus not reached

Round 2

Six items

Tool Content
- Anonymized outcomes report of Round 1 including comments
- Six items (#2-7) not achieving consensus from Round 1

→

Outcomes

Item 2. Consensus reached (81.3%)

Item 3. Consensus reached (87.5%)

Items 4-7. Consensus not reached

Round 3

Four items

Tool Content
- Anonymized outcomes report of Round 2 including comments
- Four items (#4-7) not achieving consensus from Round 2

→

Outcomes

Item 4. Consensus not reached. Participants frequently commented that item was too long because they could see all three potential sub-questions. Item was kept in the final version as participants would only see the sub-questions triggered by their response.

Item 5. Consensus not reached; but kept item for testing phase given inconsistency in feedback.

Item 6. Consensus not reached. However, PCPs commented they would be willing to share this if requested by the specialist. In response, the research team added a similar question to the specialist RLT in the third round.

Item 7. Consensus not reached. Conflicting responses by PCPs. Some raised concerns that a working relationship could be damaged if a specialist felt the feedback was negative. Others felt that it was important to provide feedback to specialists. The research team decided to keep the item, making it optional.
