# Supplementary material for: Development of eConsult reflective learning tools for healthcare providers: a pragmatic mixed methods approach
Source: BMC Prim Care. 2023 Jan 16;24:15. doi: 10.1186/s12875-022-01948-9 (PMC9841624; doi:10.1186/s12875-022-01948-9)
Supplement: Supplementary file 2 — Additional file 2. Results of the three Delhi rounds for specialists’ reflective learning tool. [file 12875_2022_1948_MOESM2_ESM.pdf]

Additional File 2. Results of the three Delhi rounds for specialists' reflective learning tool

Round 1

Five items including open text boxes for comments

Tool Content

Item 1. Was the clinical question clear?
- Option yes or no.
- If yes, four response options (select all that apply)
- If no, additional yes or no question

Item 2. Did the PCP include sufficient and appropriate information to facilitate your consultation?
- Option yes or no.
- If no, six response options (select all that apply)

Item 3. Did you use any additional resources to answer the PCP's question other than personal knowledge/experience?
- Option yes or no; if yes then 6 response options (select all that apply)

Item 4. Did you seek additional information from the referrer?
- Option yes or no
- If yes, additional two checklist questions

Item 5. Did you learn anything from this eConsult request?
- Option yes or no
- If yes, indicate one learning point
- If no, question ends

Item 6. How are you planning to use this information in your practice?
- Open text field.

→

Outcomes

Item 1. Consensus reached (81.75%)
Item 2. Consensus reached (93.75%)
Item 3. Consensus not reached
Item 4. Consensus reached (87.5%)
Item 5 and 6. Consensus not reached, combined items for next round.

Round 2

Two items

Tool Content

- Anonymized outcomes report of Round 1 including comments
- Two items (#3 and #5) not achieving consensus from Round 1
- **Item 5.** Did you learn anything from this eConsult request?
  - If yes, then previous Item 6 question "How are you Planning to use this information in your practice?"
  - If no, questions ends

→

Outcomes

Item 3. Consensus not reached
Item 5. Consensus not reached

Round 3

Three items

Tool Content

- Anonymized outcomes report of Round 2 including comments
- Two items (#3 and #5) not achieving consensus from Round 2
- Question added:

Item 6. Would you like the PCP to share the patient outcomes with you?
- Checkbox option

→

Outcomes

Item 3. Consensus not reached
Item 5. Consensus not reached
Item 6. Consensus not reached
