# Supplementary material for: Development of eConsult reflective learning tools for healthcare providers: a pragmatic mixed methods approach
Source: BMC Prim Care. 2023 Jan 16;24:15. doi: 10.1186/s12875-022-01948-9 (PMC9841624; doi:10.1186/s12875-022-01948-9)
Supplement: Supplementary file 3 — Additional file 3. PCP RLT Algorithm. [file 12875_2022_1948_MOESM3_ESM.pdf]

Additional File 3. PCP RLT Algorithm

1. Contact Information

Name:

Email address:

2. What Was Your Clinical Question?

Question Type: (Select all that apply)

- ☐ Clinical management
- ☐ Diagnostic
- ☐ Treatment
- ☐ Other:

Specialty or specialties consulted:

3. Learning

How did the specialist's response to this eConsult impact your knowledge or understanding? (Select all that apply)

- I learned something new
- I am motivated to learn more
- I intend to use this information in my teaching
- I am reassured
- I am reminded of something I already knew
- This response will not impact my knowledge and understanding

Please enter at least one learning point.

4. Improvements to the Specialist's Response

Could this response have been improved?

- No
- Yes

What problems did you see? (Select all that apply)

- ☐ Did not answer the question I posed
- ☐ Too much information
- ☐ Not enough information
- ☐ Information poorly written
- ☐ Too technical
- ☐ Other

5. Application to Patient Care

Will you use this eConsult information for your patient?

- Yes
- Possibly
- No

As a result of this information, I will/may… (Select all that apply):

- ☐ Manage this patient differently
- ☐ Use this information to justify a choice
- ☐ Use this information to be more certain about the management of this patient
- ☐ Use this information to better understand a particular issue related to this patient
- ☐ Use this information to inform my discussion with the patient or other healthcare professionals.

I will not use this information because… (Select all that apply):

- ☐ I disagree with the content of this information
- ☐ The advice does not align with the patient's preferences
- ☐ The response will not affect my practice
- ☐ This information is potentially harmful
- ☐ I am unable to act on the information provided
- ☐ I do not trust the information provided

Please elaborate on how this information is potentially harmful

Please elaborate as to why you are unable to act on this information

Please elaborate on why you do not trust this information

6. Sharing Patient Outcomes

If you and this patient are willing to share the patient outcomes with the specialist, please click here.

7. Are you willing to share a copy of this survey with the specialist?

- Yes
- No

Submit
