# Supplementary material for: Development of eConsult reflective learning tools for healthcare providers: a pragmatic mixed methods approach
Source: BMC Prim Care. 2023 Jan 16;24:15. doi: 10.1186/s12875-022-01948-9 (PMC9841624; doi:10.1186/s12875-022-01948-9)
Supplement: Supplementary file 4 — Additional file 4. Specialties or Services consulted by PCPs. [file 12875_2022_1948_MOESM4_ESM.pdf]

Additional File 4. Specialties or Services consulted by PCPs

Specialties	Champlain BASE	SFHN eReferral	Total
Internal Medicine Specialties (Cardiology, Endocrinology, Haematology, etc.)	15	12	27
CCM (Complex Care Management)	0	3	3
Dermatology	7	0	7
ENT	0	1	2
Neuropsychology	0	2	2
Surgical Specialties (Neurosurgery, Obstetrics/Gynaecology, Orthopaedics, etc)	5	11	16
Paediatrics	1	0	1
Pain	1	0	1
Physiatry	0	1	1
Radiology	0	2	2

Note: Not all specialities or services listed here are offered by both eConsult systems.
