# Supplementary material for: Development of eConsult reflective learning tools for healthcare providers: a pragmatic mixed methods approach
Source: BMC Prim Care. 2023 Jan 16;24:15. doi: 10.1186/s12875-022-01948-9 (PMC9841624; doi:10.1186/s12875-022-01948-9)

Additional File 5. Specialist RLT Algorithm

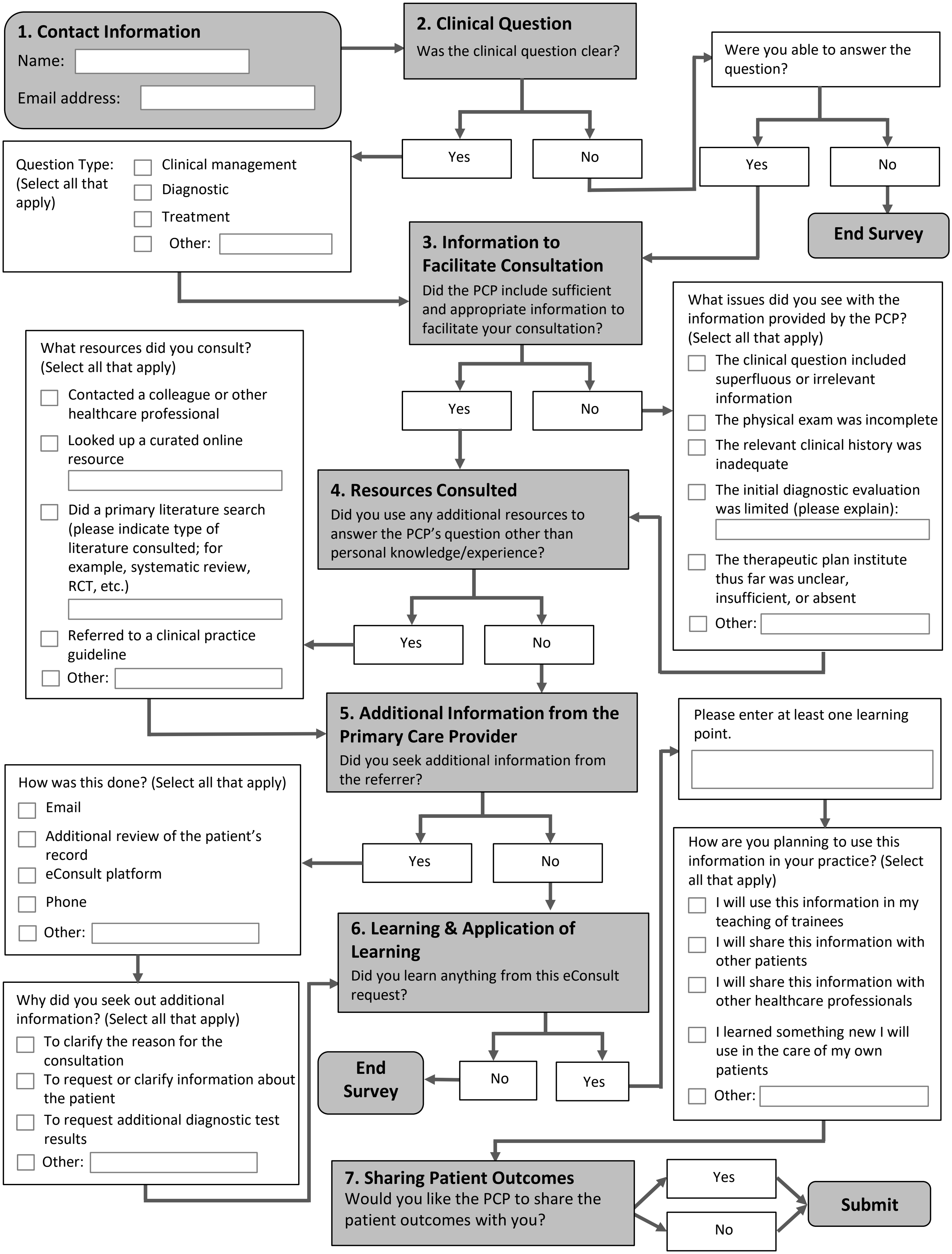

Supplement: Supplementary file 5 — Additional file 5. Specialist RLT Algorithm. [file 12875_2022_1948_MOESM5_ESM.pdf]
